# Special Issue: Recent Developments on High-Performance Fiber-Reinforced Concrete: Hybrid Mixes and Combinations with Other Materials

**DOI:** 10.3390/ma15093409

**Published:** 2022-05-09

**Authors:** Carlos Zanuy

**Affiliations:** Department of Continuum Mechanics and Structures, Universidad Politécnica de Madrid, ETS Ingenieros de Caminos, c/Profesor Aranguren 3, 28040 Madrid, Spain; carlos.zanuy@upm.es

## 1. Introduction

The use of high- and ultra-high-performance fiber-reinforced concretes (HPFRC and UHPFRC, respectively) has increased significantly in the last few years as a result of large research efforts and collaboration between research and industry. Among the most recent developments aiming for the optimization of the material’s possibilities, researchers have tried to combine different fiber types within the cementitious mix, including different fiber configurations or materials, in order to understand and take advantage of the mechanical interaction among the mix constituents [1,2,3]. New advancements regarding the aggregates, cement or additives have favored the development of engineered composites with optimized particle-packing, water-reducing agents and curing methods [4,5]. Special mixes have been developed which perform satisfactorily under severe load conditions and environments such as fatigue [6], impact [7] or corrosion [8]. In addition, HPFRC and UHPFRC have been combined with other materials to form composite members or strengthen and retrofit existing structures. Research challenges such as the bonds between materials or the interaction of rheological effects [9] must be addressed for such composite elements.

The combined performance of FRC or UHPFRC with other materials relies on the mechanisms governing the compatibility and the eventual slip development between the materials. The characterization of interfacial surface roughness is essential to understand the mechanics between concretes, including adhesion, friction or interlocking components [10]. Specific bond–slip models have been reported for the embedment of UHPFRC in reinforcing steel, fiber-reinforced polymer (FRP) bars and prestressing strands [11,12,13], showing an appreciable bond strength increase with respect to concrete-embedded members. An interesting pseudo-ductile bond–slip performance has been even observed between UHPFRC and steel, which cannot be achieved between conventional concrete or HPFRC and steel [5], thus requiring additional mechanical connectors. The partial mechanical interaction between combined materials makes it challenging to design composite structures, and conventional strain-compatibility-based models must be abandoned to include relative slip development. The appropriate understanding of composites’ performance is necessary even for the implementation of simplified methods based on the reference length concept, as suggested by [14] for smeared crack localization. The compatibility of extension conditions, as proposed by [15,16], is more susceptible to the influence of crack localization, but the consideration of real adherence or bond mechanics is necessary. For this, specific studies such as those presented in this Special Issue are required. It is expected that the high-quality research presented in this Special Issue can contribute to a step forward towards the combined use of HPFRC and UHPFRC with other structural materials.

## 2. Short Description of the Papers Published in the Special Issue

The objective of this Special Issue is to gather the most recent and relevant research results from materials, structural, chemical and mechanical engineering experts in this field. Research works at both the material and structural level are presented in this Special Issue.

At the material level, Małek et al. [17] investigated how the material properties of fiber-reinforced mortars can be affected by the use of recycled fibers from melted glass waste from bottles. The research is a significant step towards the sustainable use of recycled components in high-performance structural materials. In turn, Paredes et al. [18] completed a study on matrix optimization for ultra-high performance concrete, without and with steel fibers, in order to enhance the material and durability properties. The study dealt with the influence of silica fume, metakaolin and nano silica, among other components.

At the structural level, four papers are presented in order to understand the interaction of FRC and UHPFRC with other materials. Zanuy et al. [19] presented an experimental and theoretical study concerning the composite behavior of tension members consisting of a reinforced concrete core strengthened with thin HPFRC layers. The study focused on composite action, including the stress transfer between the different materials through bonds. The interesting topic of tension stiffening was dealt with in the paper, and the influence of the shrinkage of the different concretes involved in the composite member was studied.

Smith-Gillis et al. [20] focused on the strengthening of ultra-high-performance concrete panels with external layers of fiber-reinforced thermoplastic composites in order to improve the strength and performance of the material against impact loads. Different bonding methods were studied in order to achieve a composite design with enhanced impact resistance.

Dudek et al. [21] dealt with the pull-out strength of steel expansion anchors embedded in FRC. This is an interesting contribution concerning the composite use of FRC and structural steel anchors. The influence of the fiber content of the FRC mix was studied for both normal- and high-strength concrete matrices. In addition, the paper accounted for the eventual presence of cracks in the embedding substrate.

As an additional contribution to the analysis of bond performance, Barkhordari et al. [22] researched the efficiency of data-driven hybrid machine-learning models for the estimation of the bond strength between fiber-reinforced polymer (FRP) laminates and concrete. The combined use of artificial neural networks and the Runge–Kutta optimization algorithm was proven to be an efficient tool to determine bond strength as a function of the geometrical and material properties of a joint. Furthermore, the simulations indicate that the employed algorithms could be further exploited for use in other bond-related problems.

## 3. Conclusions

The papers published in this Special Issue of *Materials* demonstrate the high level of interest from researchers in the field concerning the combined use of FRC and HPFRC with other construction materials, as well as the need for an optimal mix design of the composite. With the present SI, it is hoped that the readers will learn about relevant contributions for their research and professional applications.
